# Experiences of parents of children with mental illnesses: A systematic review and meta‐ethnographic synthesis

**DOI:** 10.1111/famp.13087

**Published:** 2024-11-24

**Authors:** Suzanna Greally, Jeppe Oute, Susan McPherson

**Affiliations:** ^1^ School of Health and Social Care University of Essex Colchester UK; ^2^ Department of People and Technology Roskilde University Roskilde Denmark

**Keywords:** caregiver burden, literature review, mental illness, meta‐ethnographic synthesis, qualitative research

## Abstract

In this paper, we present a qualitative meta‐ethnographic synthesis of 26 articles reporting experiences of subjective burden in 389 parents of children with mental illnesses. The studies were identified through a systematic literature search of PubMed, CINAHL Ultimate, APA PsycInfo, and MedLine Ultimate. We conducted a quality appraisal and followed the seven stages of meta‐ethnographic synthesis. We developed a line of argument synthesis in the form of a model that depicts the subjective, temporal, and psychological experiences of parents. The model proposes a cyclical process characterized by five stages, each depicting a transitional point in which parents re‐evaluate their experiences: caring for an intimate stranger; turning point; unavoidable role; uncertain future; transcendence. This model highlights the complex psychological processes that parents endure when caring for a child with a mental illness. The review highlights several methodological issues in this field of research, including issues related to sampling, poor reporting of data analysis, limited critical appraisal, and a lack of reflexivity. Future research should address the gaps in the literature while also attending to the issues that have been highlighted by this review.

Literature has highlighted the many difficult experiences of family caregivers, with the term *caregiver burden* often used to describe these experiences. This term was first documented in the 1980s to describe the financial, social, emotional, psychological, and physical consequences of caregiving (George & Gwyther, 1986). Since then, caregiver burden has been defined through the categories of objective and subjective burden. Objective burden denotes the *caregiving labor* (Oute & Huniche, 2017) and objective changes to, for instance, routine, work, income, leisure time, hobbies, and lost career opportunities (e.g., Kumar & Gupta, 2014). Subjective burden encompasses the psychological consequences for the caregiver (Schene, 1990) and is thought to be the most important factor affecting caregiver well‐being (del‐Pino‐Casado et al., 2021).

Caregiving and caregiver burden may be experienced differently across different cultures, with diverse meanings attributed to caregiving and differing cultural norms for the expression of caregiver burden; some of these aspects are briefly considered here. Religion may play a role; Lim et al. (1996) described how Mexican Americans value the ability to endure suffering and consider caregiving as a fate that ensures a place in heaven. Hebert et al. (2006) summarized how a supportive social network, coping resources, and positive emotions are some of the aspects of religion thought to improve caregiver well‐being. Ethnicity may also be important; in a study conducted in the United States of America (USA), Filipinos were more likely to emphasize how they learned from their caregiving experiences than non‐Hispanic White caregivers (Ivey et al., 2013). Policies and guidelines may also impact caregiving; research into the responsibilization of caregivers in the United Kingdom and Denmark (McPherson & Oute, 2021) highlights how depression guidelines in these countries reflect an objective of relieving the burden of illness on the state through the responsibilization of family caregivers. However, it seems there is no consensus on how culture mediates the caregiving experience. While some studies report important differences, it has also been argued that the experience of caregiving may be “its own cultural entity,” superseding race or ethnicity (Siefert et al., 2008, p. 399).

There are significant consequences to the well‐being of those living with and/or providing care to family members, including those with a physical illness, mental illness, or neurocognitive disorder. The experience of living with a relative with dementia has been described as an “ongoing funeral” (Kapust, 1982, p. 79), and more recent research shows that the trajectory of caregiver burden in dementia is to increase over time (Van den Kieboom et al., 2020). Relatives living with an adult with depression experience instability in their everyday lives and have little support from others (Ahlström et al., 2009), and the impact of caring for a partner with depression is so significant that this disorder has been termed a “couples' disease” (Priestley et al., 2018, p. 128).

While there has been some research into caregiving experiences within mental health, the tendency has been to focus on spousal and other adult‐to‐adult caregiving relationships (Priestley & McPherson, 2016). Given that a strong sense of loyalty in the relationship (Priestley et al., 2018) and co‐habitation (Van den Kieboom et al., 2020) are hypothesized to increase caregiver burden, one might expect parents to experience high levels of caregiver burden in relation to caring for a child with a mental illness. Despite this, there is little research on the experiences of these parents, as the existing literature has concentrated on physical illnesses and disabilities. Furthermore, existing research on parental caregiving has generally focused on objective burden, such as views on medication (e.g., Lazaratou et al., 2007), the barriers and facilitators to seeking psychological therapies for a child (e.g., Reardon et al., 2017), and other practical experiences regarding attempts to access services for a child (e.g., Crouch et al., 2019). Comparatively less attention has been given to contextualized depictions of subjective experiences of burden. Research concerning parents of children with chronic physical disabilities suggests that these parents experience high levels of stress (e.g., Cousino & Hazen, 2013), and parents of children with intellectual disabilities experience conflict, exhaustion, and fear for the future (Willingham‐Storr, 2014). However, research involving parents of children with mental illnesses appears to be more limited, and this literature has not previously been systematically reviewed or synthesized. This highlights an important gap in the literature: little is known of the experiences of subjective burden in the parents of children with mental illnesses.

The role of parents in the treatment and management of mental illnesses in their children is critical, from the recognition of problems through to access to care, adherence to treatment, and treatment outcomes (Mackova et al., 2022). However, if their experiences are not well understood, it is difficult to provide them with adequate support. Given the likelihood of subjective burden, which may impact the care they are able to provide, parents need to be appropriately supported to care for their children, whose needs and difficulties can be complex. A review of the existing literature on this subject may reveal a new understanding of the experiences of these parents and lead to useful developments in clinical practice, improving care for both parents and children.

## Aim

This paper aimed to review the literature on the subjective experiences of parents of children with mental illnesses. Given that the body of research on the topic is small, this review included parents who were caring for children of any age who were receiving treatment for a mental illness in any treatment setting. Rather than producing a broad overview of existing research, the aim was to produce a synthesized understanding of the evidence in a way that contributed to theoretical development and clinical practice by drawing on carefully selected studies, including a quality appraisal, and using a methodology that allowed for both flexibility and rigor. These aims indicated that a systematic review was a more appropriate choice than a scoping review (Munn et al., 2018). This paper presents a qualitative systematic review and meta‐ethnographic synthesis, led by the question: what are the subjective experiences of parents who care for a child with a mental illness?

## METHOD

### Search strategy

We conducted a systematic review of peer‐reviewed studies and dissertations that reported research on primary caregivers (primarily parents) of children of any age with mental illnesses using qualitative methodology. We searched four databases (PubMed, CINAHL Ultimate, APA PsycInfo, and MedLine Ultimate) using synonyms of “child,” “mental disorders,” “parent,” and “qualitative.” English language was used as a limiter. This search, conducted in June and July 2022, yielded 2494 references; these were imported into EndNote (https://endnote.com) and 244 duplicates were removed. This search was updated in February 2024; 83 additional records were screened.

#### Inclusion and exclusion criteria

**FIGURE 1:**
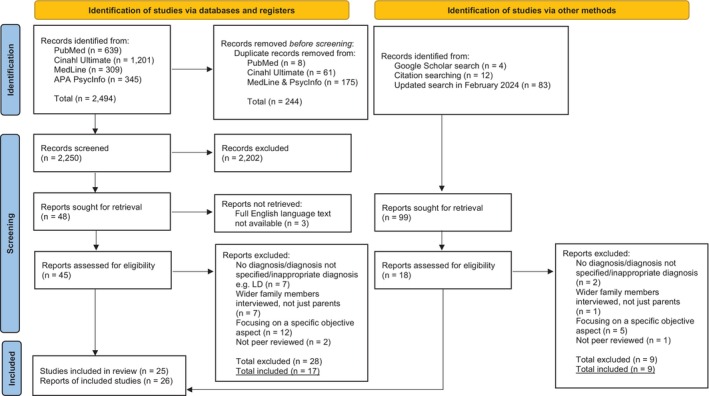
PRISMA flow diagram.

Our review included journal articles and dissertations that used a qualitative approach to explore the subjective experiences of parents who have a child with a mental illness. The exclusion criteria were: (a) studies in which the child did not have a formal diagnosis of a mental illness, (b) research concerning caring for a child with a diagnosis of Learning Disability, Tourette's Syndrome, Attention‐Deficit/Hyperactivity Disorder, or Autism Spectrum Disorder without co‐morbid mental illness; (c) research with mixed samples, in which interviews were conducted with non‐primary caregiver family members (e.g., siblings, partners), rendering it impossible to separate the findings and conclusions regarding parental interviews from other family members; (d) studies that focused solely on objective experiences or specific aspects of care (e.g., medication, parental beliefs about cause of illness, barriers/facilitators to accessing treatment, transition to adult care, professionals' ways of working with parents, help‐seeking, receiving diagnosis, and evaluation of specific treatment program), rather than subjective experiences. Articles were screened against the eligibility criteria: 2205 studies were excluded after reading titles and abstracts, 45 full‐text papers were evaluated, and 17 relevant studies were identified. When the search was updated in February 2024, two additional studies were identified and included. Finally, forward and backward reference searching of the included studies yielded an additional seven relevant studies that were included in the review. The detailed study selection process is displayed in the PRISMA flow diagram (Figure 1).

### Data synthesis

Approaches to meta‐synthesis are varied. Some researchers employ the methods used in thematic analysis (such as line‐by‐line coding) to the studies to be reviewed, as in thematic synthesis (Thomas & Harden, 2008). Another approach is that of meta‐narrative review (Greenhalgh et al., 2005), which allows for the appraisal of studies of similar phenomena that have produced seemingly contradictory results. Meta‐ethnographic synthesis was initially purposed for the reviewing of ethnographies but was later deemed appropriate for qualitative research beyond ethnography (Britten et al., 2002; Thomas & Harden, 2008). In the present study, we followed the seven stages of meta‐ethnographic synthesis outlined by Noblit and Hare (1988): getting started, deciding what is relevant to the initial interest, reading the studies, determining how the studies are related, translating the studies into one another, synthesizing translations, and expressing the synthesis.

A feasibility study by Campbell et al. (2003) demonstrated the effective use of a meta‐ethnographic approach in synthesizing research that covers care in the context of chronic illness, having noted that meta‐ethnographic synthesis is thought to be the best‐developed method for synthesizing qualitative data. The authors concluded that qualitative studies can be synthesized helpfully and successfully by using a meta‐ethnographic approach in order to provide novel ways of understanding a phenomenon. They noted that this approach is likely to offer greater insight and theoretical development than other approaches, such as meta‐narrative reviews. Furthermore, meta‐ethnography is used in particular for reviews addressing the meanings or processes of phenomena (Gough et al., 2012) and is considered useful for addressing experiences of illness and care (Priestley & McPherson, 2016). It is also considered by some to be the most well‐developed qualitative synthesis method (e.g., Britten et al., 2002).

The results of meta‐ethnographies will differ depending on the author, and, as Dixon‐Woods et al. (2006) highlighted in the development of their new approach to meta‐ethnography, it is therefore important to ensure that conclusions are grounded in the evidence while maintaining reflexivity during the interpretative work required in synthesizing qualitative studies. The searches and data synthesis were performed by the first author, who has an academic and clinical interest in childhood mental health and caregiver burden. She has worked in various National Health Service (NHS) organizations in the United Kingdom (UK), including community and inpatient mental health services for children/adolescents and a family therapy service. The searches and synthesis were performed with oversight from, and in discussion with, two researchers experienced in qualitative health research and meta‐ethnography. The first author kept a reflexive journal throughout the process, consulted with experienced colleagues, employed a systematic search strategy, and conducted a critical appraisal of the included studies. She followed PRISMA guidelines to promote transparency and rigor. The steps taken during the synthesis are detailed below.

The first author organized the studies chronologically by publication date and completed an initial reading of each, starting with the oldest (Campbell et al., 2003). She then re‐read the results and discussion sections. She made annotations, particularly paying attention to the themes devised by the original authors, the theories described, information directly related to the research question of this review, and concepts that appeared to be recurring across the studies. On a subsequent re‐reading of each study, she extracted and tabulated key information relating to methodology, adding a new column for each study. Next, by re‐reading the results and discussion sections and the handwritten annotations, she was able to detect ideas, theories, or themes that were present across several studies; she identified these as key concepts that were common across the studies. During the process of identifying key concepts, she continuously compared the studies against these, and both excluded concepts and added new concepts based on this constant comparison. This produced a final set of 10 key concepts; these were added to the table.

The first author then re‐read the results and discussion sections of each study with the key concepts in mind, allowing for data to be extracted relating to each of the key concepts and added to the table. This process made it possible to uncover each concept described in each study and ensure that the initial concepts in each study were captured by the new key concepts that she had developed and tabulated. In completing the table, she preserved the original authors' language as much as possible in order to remain faithful to the original studies (Britten et al., 2002) and to reduce the likelihood of re‐interpreting the original authors' concepts. She added a final row to the table, containing second‐order interpretations (Schutz, 1962, as cited in Britten et al., 2002), which go beyond lay‐person understanding and illustrate the principal theories or explanations of each study, often drawing on psychological and social science theory. In this row of the table, quotation marks were used to denote use of the authors' own words, as in Britten et al.'s (2002) paper.

During the process of adding information from each study into a new column in the table, it started to become possible to see how many of the key concepts featured in each study. To better facilitate this cross‐comparison of studies by concept, the first author devised a new table, illustrating how the key concepts were distributed across the 26 studies (see Table S1). This made it easier to establish that the studies were not refutational of one another; the commonalities across the studies and their key concepts suggested reciprocal relationships. Moreover, by continuing to re‐read the key concepts and translate these across the studies, it was possible to identify similarities and overlap between some of the key concepts. The 10 key concepts were synthesized into five final concepts (see Table 1), depicting broader themes. These broader themes were possible to develop only after the cross‐comparison and synthesis of the studies, and these themes provide an interpretation that goes beyond the findings of each individual study.

**TABLE 1:** Key concepts and synthesized concepts.

Key concepts	Final synthesized concepts
Transformation of the loved child	Caring for an intimate stranger
A psychological tsunami of emotions	
Realization	Turning point
Getting help	
Gendered caregiving	Unavoidable role
Commitment	
An impossible task	
Uncertain future	Uncertain future
Reframing thinking	Transcendence
Finding meaning	

## RESULTS

### Characteristics of the studies

The 26 studies included in this meta‐ethnographic synthesis capture the experiences of 389 parents (279 mothers and 110 fathers). The studies were conducted across 12 countries (Canada, China, Greece, Ireland, Oman, Scotland, Spain, Sweden, Thailand, Turkey, the United Kingdom, and the United States). The children had a range of diagnoses, but there was a disproportionate focus on schizophrenia or other psychotic disorders, with 13 of the studies focusing solely on these disorders. The remaining studies encompassed a range of diagnoses, including, but not limited to, eating disorders, depression, and bipolar affective disorder. Nevertheless, the results are likely to reflect a bias toward psychotic disorders, potentially overshadowing experiences associated with other diagnoses.

Many researchers failed to make clear the inclusion and exclusion criteria relating to the participants' children. In several studies, it was unclear from the recruitment and sampling strategies whether the children were currently receiving treatment. The context of treatment was also not reliably described; a small number of studies specified that the children were living at home and/or receiving community treatment, while three studies specified that participants were recruited via both inpatient and community settings. One study stated that the children were in a “day hospital” (Sarrió‐Colas et al., 2022, p. 45), and another stated the children were receiving “intensive specialist treatment” (Svensson et al., 2013, p. 396); it was not clear what these terms meant. Nine studies did not provide details of the treatment setting. The consequence of apparent leniency regarding inclusion/exclusion criteria is that it is likely that within many of the studies included in this review, parental experiences were amalgamated, despite vastly different circumstances; parents of children who receive community treatment have different experiences to those of children who receive inpatient treatment.

Studies differed in how they grouped the children by age, and, in general, researchers did not provide a clear rationale for their choice of age group. Some studies focused on children under the age of 18 years (e.g., aged 6–11 years; Wade, 2006) or adult children over the age of 18 years (e.g., aged 18–35 years; Wiens & Daniluk, 2009). Others grouped children differently, such as aged 15–19 years (Al Yahyaei et al., 2024) or 17–31 years (Mohr & Regan‐Kubinski, 2001). In many of the studies concerning adult children, the ages varied widely; it would be problematic to assume that parents of a 16‐year‐old have equivalent experiences to parents of a 37‐year‐old, and yet, in many of the studies, differences in age and life stage were not discussed. On the whole, in 11 studies the children were adolescents or younger, in 13 studies the children were adults, and in two studies this information was not provided.

### Quality appraisal

Despite debate on the matter, many authors consider quality appraisal to be an essential part of qualitative meta‐synthesis (e.g., Carroll et al., 2013). The Critical Appraisal Skills Programme tool for qualitative research (CASP, 2018) was developed for use within health research and is recommended by the Cochrane Qualitative and Implementation Methods Group. The CASP tool was used to appraise the studies included in this review. As it is not clear whether, or how, lower quality studies should be excluded (Dixon‐Woods et al., 2006), all eligible studies were included in the review.

A number of limitations were highlighted by the quality appraisal. Issues related to sampling have already been described, and the results of this review should be considered in light of these. Further issues included poor reporting of data analysis, limited critical appraisal, and a lack of reflexivity. These cast doubt on the validity of the existing research. However, given the dearth of literature on the topic, posing further constraints on the studies (by means of additional exclusion criteria) would have led to a much smaller body of research to review, making it difficult to draw any meaningful conclusions about parental experiences. All studies were therefore included in the review, but additional limitations highlighted by the quality appraisal are discussed below.

Five studies did not provide sufficient information about data analysis to determine how the analysis was undertaken, including how themes were derived from the data. For example, Donnelly (2001) stated that data from the interviews were “synthesised into common themes” (p. 294), with no details provided as to how this was achieved. This author described conducting a “unique method of data analysis,” (p. 294) but did not offer a description of their methodology, meaning the analysis cannot be replicated. In another study (Akgül Gök & Duyan, 2020), the authors used thematic analysis but did not comment on the underlying assumptions underpinning the analysis, and it was not possible to determine what type of thematic analysis they performed.

Eight studies did not describe whether, or how, the relationship between researcher and participants had been considered, meaning that it was unclear whether researchers had thought critically about their own influence on the research. Several studies commented on themes emerging from the data, whereas Braun and Clarke (2021) advise against the use of this passive language as it does not acknowledge the active researcher role in constructing themes. Mohr and Regan‐Kubinski (2001) cited measures of trustworthiness that they used to address the validity and credibility of their research. However, in describing their data analysis, they stated that transcripts were “analysed for patterns and trends” and “themes […] were negotiated with professional colleagues” (p. 71) without any indication of what method of analysis was used or how themes were developed. It was also not clear what the negotiation of themes involved, and thus it would not be possible for other researchers to attempt to analyze data in a similar way, or assess whether the author's methods did enhance the trustworthiness or rigor of the study.

Of the studies that did describe how the relationship between researcher and participants had been considered, there were varying degrees of detail. Better quality studies described reflexivity on the part of the researcher, with consideration given to how both the dialogic nature of interviews and the characteristics of the interviewers might have affected the information shared by participants and therefore the results of the studies. Harden (2005a), for instance, commented on the “reflexive process” (p. 210) of the analysis and considered how the participants told stories as well as reconstructed stories through the active process of engaging in their interviews.

The results of the review are presented below.

### Caring for an intimate stranger

#### Transformation of the loved child

The phrase “transformation of the loved child” (Tuck et al., 1997, p. 121) came from a theme in a study involving parents of adult children with schizophrenia in the United States, but the concept featured in almost every study. As the child became ill, parents witnessed a “malevolent transformation of a loved one who remains physically present in their world” (Tuck et al., 1997, p. 123), reminiscent of Boss' (2002) concept of *ambiguous loss*. The significant changes that parents noticed in their child required them to parent their child differently. This was described as “parenting in overdrive” in a study involving parents of adolescent children with depression in the United Kingdom (Stapley et al., 2016, p. 624), as the child, rather than developing independence, became more dependent with age. The extent of this change was captured in a study of parents of adolescent children with eating disorders in Sweden, in which parents had to behave as though caring for a toddler rather than their late‐teenage child (Svensson et al., 2013).

The studies captured how parents perceived their child to be significantly different as a consequence of their illness, rendering the child almost unrecognizable at times. This featured in studies involving parents of adult children as well as young children. In one study of Thai mothers caring for an adult child with schizophrenia, mothers thought their child looked “like a stranger” or as though they were possessed (Kanungpiarn et al., 2021, p.176). The experience of simultaneously recognizing and not recognizing was captured by Darmi et al. (2017), who described how Greek parents of adult children with psychosis were “caring for an intimate stranger” (p. 197).

Parents of adult children with psychosis had some differing experiences. For many, there was a sense that the changes within their child were so profound that there was “no going back” (Mohr & Regan‐Kubinski, 2001, p. 76) and no way to restore the “pre‐illness normal state” of their child (Poonnotok et al., 2016, p. 79). Some parents, however, perceived there to be “a core identity” of the child that was unchanged (Tuck et al., 1997, p. 121), enabling them to “glimpse” the pre‐illness child at times (Pejlert, 2001, p. 199; Tuck et al., 1997, p. 123), engendering hope.

Most studies described feelings of loss over the transformation of the child. One study of UK mothers' experiences of having an adolescent child with depression conveyed this with the theme “depression causes change” (Armitage et al., 2020, p. 1623). This encompassed the loss of connection with the child, the loss of the child's personality, and the loss of normal family life. In some studies, the profound effect of the transformation of the child caused parents to feel as though the child was not only changed but gone entirely. Parents of adolescent children with various mental illnesses in Spain wanted to “get back” their lost child (Sarrió‐Colas et al., 2022, p. 50), and the sense of loss, experienced by Swedish parents of adult children with various mental illnesses, created feelings of grief and “constant sorrow” (Johansson et al., 2010, p. 695).

#### A psychological tsunami of emotions

McAuliffe et al. (2014) used the metaphor of a “psychological tsunami” (p. 148) to describe the devastation experienced by parents. Parents experienced emotions including (but not limited to) shock, fear, grief, guilt, anger, and helplessness, which began at the time of noticing changes in their child, and were often compounded at the point of diagnosis. The painful emotions were unrelenting during the course of the child's illness, meaning that parents experienced “suffering as a way of life” (Donnelly, 2001, p. 297), and learned to “live with a constant sadness” (Johansson et al., 2010, p. 696). When considered together with the grief and mourning described in the previous paragraph, this is suggestive of Olshansky's (1962) concept of *chronic sorrow*.

Many parents worried about their own culpability in their child's illness, causing them to question their ability as parents. Scottish parents of adolescent children (with various mental illnesses) felt shame and regret and were tormented by guilt, particularly at the beginning of their child's illness (Harden, 2005b), believing themselves to be failing as parents (Harden, 2005a). Feelings of uncertainty and powerlessness created a “disconcerting emptiness” (Mohr & Regan‐Kubinski, 2001, p. 73), and experiences of stigma and judgment exacerbated parents' struggles. This was described as “distressing loneliness” in a study involving parents of pre‐adolescent children with bipolar affective disorder in the United States (Wade, 2006, p. 895). For some parents, matters were further complicated by a belief that they had to conceal their emotions (Armitage et al., 2020) or attempt to uphold positive feelings (Kanungpiarn et al., 2021) in order to help their child. This was perhaps due to the stigmatized nature of some of the emotions experienced by the parents, including anger and resentment (Kalayci et al., 2023). The complexity and intensity of emotional experiences was apparent across the studies, with the array of difficult emotions described as “turmoil” in studies conducted both in the United Kingdom (Stapley et al., 2016, p. 622) and the United States (Raymond et al., 2017, p. 125).

Emotional turmoil was evidently a prominent aspect of parents' experiences, such that the central themes in one study of Swedish mothers' experiences were “mourning, loss, and the sense of a never‐ending burden” (Piuva & Brodin, 2020, p. 1025). The concept “a psychological tsunami of emotions” featured in all but two of the studies; in the two studies in which this concept was not present, the results sections were brief and did not provide detail on the emotional experiences of the parents, who were caring for adolescent children with various mental illnesses in Spain (Sarrió‐Colas et al., 2022) and adolescent children with anorexia in the United Kingdom (Thomson et al., 2014).

### Turning point

#### Realization

In the majority of studies, the authors described moments when parents came to a realization regarding their child's difficulties. For most, this acknowledgement of the extent of their child's difficulties occurred either at the time of recognizing the child's symptoms, after an acute episode of psychological distress, or at the time of diagnosis. Mohr and Regan‐Kubinski (2001) described how this “ranged from a gradual perception to a sudden realisation” (p. 72).

These important moments of realization for families were crucial in the narration of events, with time seemingly “sharply divided” (Tuck et al., 1997, p. 120) into life before and life after the onset of illness or the diagnosis. Experiences of diagnosis varied. Receiving a diagnosis of bipolar disorder for a pre‐adolescent child caused relief (Wade, 2006) when it had been preceded by previous misdiagnoses, incorrect medication, and a strong sense that something was wrong. Similarly, diagnosis provided a decrease in anxiety for mothers of adult children with various mental illnesses in Sweden because it facilitated an improved understanding of the child's difficulties (Johansson et al., 2010). However, the majority of studies described intense, often negative, emotions connected to receiving a diagnosis; schizophrenia, in particular, appeared to be received negatively and incited significant concern in the parents. Parents in China experienced this diagnosis as overwhelming and hard to accept (Bai et al., 2020), and Canadian parents experienced “devastation” as a result of the diagnosis (Wiens & Daniluk, 2009, p. 343). Parents in Thailand perceived a diagnosis of schizophrenia to mean “facing shattered dreams” (Poonnotok et al., 2016, p. 75).

In other studies, a moment of realization came only after traditional healing methods had been tried and failed. In Donnelly's (2001) study involving Korean American parents of adult children, the moment of realization was a point of crisis reached when “traditional” or “folk” (p. 295) healing methods had been ineffective, giving them no option other than to pursue so‐called “Western” (p. 295) treatments (such as medication); this was conceptualized by the sub‐theme “awakening” (p. 295). Similarly, for Thai mothers caring for adult children, an important turning point occurred after they had tried combining traditional and Western treatments, as it followed that they discovered that modern medicine seemed to help their child (Kanungpiarn et al., 2021).

#### Getting help

Every study described parents' experiences of seeking and/or receiving help for their child. The help‐seeking process was often complicated, with parents not knowing how or where to obtain support (Armitage et al., 2020; Johansson et al., 2010). Nevertheless, parents searched for answers and for help, desperate to know how to support their child (Poonnotok et al., 2016; Tuck et al., 1997).

When they did access support, many were frustrated or disappointed with the help they received (Armitage et al., 2020; Gok & Duyan, 2020; Harden, 2005a, 2005b; Johansson et al., 2012; McAuliffe et al., 2014; McCormack & McCann, 2015; Mohr & Regan‐Kubinski, 2001; Pejlert, 2001; Piuva & Brodin, 2020; Raymond et al., 2017; Sarrió‐Colas et al., 2022; Stapley et al., 2016; Wade, 2006; Wiens & Daniluk, 2009). Some particular factors contributed to parents' dissatisfaction. In a study that focused specifically on fathers' experiences of caring for adult children with various mental illnesses, fathers felt ignored and often perceived healthcare professionals as taking little interest in their caregiving role (Johansson et al., 2012). Other parents felt excluded from care (Harden, 2005a, in Scotland) or treated insensitively by professionals (Gok & Duyan, 2020, in Turkey). Positive experiences of healthcare were described in only a few studies (McCormack & McCann, 2015; Piuva & Brodin, 2020; Svensson et al., 2013). It was not possible to identify whether there were particular characteristics of the samples in these three studies that would explain why these participants reported positive experiences, while participants in other studies did not. For example, these were not the only studies in the review to have been conducted in these countries (Ireland and Sweden) the ages of the children (adults and adolescents) were nonspecific, and the diagnoses (eating disorders and unspecified serious mental illnesses) also featured in other studies.

For families, the process of getting help was generally long and convoluted, described by authors as a “long journey” (Raymond et al., 2017, p. 123) or a “long road” (Sarrió‐Colas et al., 2022, p. 48). Even when help was given, this brought up complicated feelings for parents. Parents of adult children with schizophrenia who were living in residential care in Sweden experienced guilt and shame which they connected to critical comments from professionals and an uncomfortable atmosphere on the ward (Pejlert, 2001). It was noted that the parents in this study favored a psychosocial understanding of their child's distress rather than a medical model of understanding; this perhaps contributed to feelings of guilt and shame when parents wondered how their own parenting had contributed to their child's difficulties. In another study involving parents of adult children with schizophrenia or a major affective disorder in the United States, parents found that the process of getting help for their child did not provide closure; rather, they instead found that treatment reinforced their worries that their child's illness could get worse (Mohr & Regan‐Kubinski, 2001). These parents also believed that their children experienced involuntary inpatient treatment as a “breach of trust” by the parents (Mohr & Regan‐Kubinski, 2001, p. 75). Parents of adult children with psychotic disorders in Greece had changing perceptions of treatment over time, but similarly experienced professionals as judgmental, and doubted the ability of professionals to help their child (Darmi et al., 2017). In this study, treatment was understood to be medication and hospitalization; psychological treatments were not discussed. Parents viewed medication as essential but had concerns about the side effects, leaving them with a sense that treatment was “cruel but necessary” (Darmi et al., 2017, p. 198).

### Unavoidable role

#### Gendered caregiving

This concept, although it featured in fewer than half of the studies, captured important differences in caregiving between mothers and fathers and therefore was important to include in this review of parental experiences. This concept generally captured how mothers assumed the main caregiving role, but in two studies, the importance of spousal support was also mentioned (Gok & Duyan, 2020; Svensson et al., 2013). However, none of the studies suggested that fathers adopted the main caregiving responsibilities; even in a study that had a sample consisting only of fathers, the authors noted that fathers still “described the mother as carrying the heaviest burden” (Johansson et al., 2012, p. 113). Previous research has highlighted the gendered division of caregiving labor in mental illnesses such as depression (Oute & Huniche, 2017), but many of the studies in this review lacked discussion around gender roles. It is not possible to say whether this was because it was not described by parents in their interviews or because the authors did not focus on this area in their analysis and write‐up of the results.

#### Commitment

Parents differed slightly in how they appraised the experience of caring for their child. The authors of two studies remarked on how none of the parents used the word “burden” to describe their experiences (McAuliffe et al., 2014, p. 149; Wade, 2006, p. 895), while other authors did use this word to describe parental experiences, such as “a huge burden” (Darmi et al., 2017, p. 199) and “a never‐ending burden” (Piuva & Brodin, 2020, p. 1025). However, irrespective of choice of language, it was evident that parents in the studies were committed to their caregiving role, regardless of the self‐sacrifice required. The language used to describe the caregiving role conveyed how this was a role that the parents could not avoid: they were “trapped” (Darmi et al., 2017, p. 200), had “responsibility” (Wiens & Daniluk, 2009, p. 343), “no choice” (Wade, 2006, p. 892), “no escape” (Mohr & Regan‐Kubinski, 2001, p. 73), and “no hope of escaping” (Donnelly, 2001, p. 297). Parents perceived their role as unavoidable, both when considering it in the present and when imagining the future (“no matter what happened, they had to stay together with their ill children and take care of each other for the rest of their lives”; Kanungpiarn et al., 2021, p. 179). The language chosen by authors again conveyed a sense of obligation; parents never considered “abdicating” their caregiving role (McAuliffe et al., 2014, p. 150). “Caregiving as an unavoidable role” (Poonnotok et al., 2016, p. 75) captured the experiences of parents overall.

#### An impossible task

Caring for a child with a mental illness was described as an immense task. The nature of the caregiving role was such that it was impossible for parents to get it right all of the time. This led to feelings of despair, such as when fathers realized that their determination to help their child was not enough (Johansson et al., 2012) or when parents of children with eating disorders found that irrespective of how much they tried to help their child, the child would not recover (Svensson et al., 2013). Furthermore, when faced with the difficulty of their task, parents were also painfully aware of what was at stake: the “potentially disastrous effect of a weak moment” (Armitage et al., 2020, p. 1624). The result was profound feelings of helplessness, powerlessness, and uncertainty, emotively described in one study as “living a life under constant strain” (Johansson et al., 2010, p. 694).

### Uncertain future

Parents were apprehensive and concerned about their child's and their own futures and viewed the future as uncertain and out of their control. There were many descriptions of a loss of dreams (Donnelly, 2001, p. 298; Gok & Duyan, 2020, p. 253; Poonnotok et al., 2016, p. 75; Wiens & Daniluk, 2009, p. 343). Some worries about the future may have been culturally mediated; parents of children in Oman worried about the prospects of marriage for their children, particularly their daughters (Al Yahyaei et al., 2024), a worry that was not described in other studies. Other parents worried about what would happen if they could not care for their child and became “afraid of dying” (Kalayci et al., 2023, p. 3).

Overall, the future was viewed by parents in complex ways. They experienced anxiety and fear, as well as hopefulness, when thinking about the future of their child and the implications this would have on their own futures. It seemed important for parents to maintain hope and to “see light in the darkness” (Johansson et al., 2010, p. 697) as it enabled them to carry on (Tuck et al., 1997). Some parents felt that hope was fundamental to the recovery process (McCormack & McCann, 2015), while others struggled to talk about the future (Sarrió‐Colas et al., 2022). Hopefulness was difficult; some parents thought it was more important to be realistic about the future (Raymond et al., 2017) or were reluctant to entertain hopeful feelings, given that hope was considered “inseparable from the risk of disappointment” (Tuck et al., 1997, p. 122). When juxtaposed, the existence of these seemingly conflictual emotions was illustrative of the uncertainty of the futures faced by the families.

### Transcendence

#### Reframing thinking

In many of the studies, parents adjusted their expectations and reframed the way they thought about their child and the future. Parents were not always aware of this process; it seemed at times to be a conscious coping strategy that helped them to accept their situation, enabling them to continue caring for their child, while at other times, a byproduct of other strategies they were using.

Religious and spiritual practices were examples of one way in which parents were able to think differently about their experiences as a consequence of using an existing coping strategy. Prayer was used by Korean American parents and meant that they “shifted their view of suffering” (Donnelly, 2001, p. 299); similarly, religious beliefs brought Thai parents feelings of peace (Kanungpiarn et al., 2021). In Kanungpiarn et al.'s (2021) study, unlike others in this review, Thai mothers believed that their suffering was caused by wrongdoings in a past life; this religious and cultural understanding of their current difficulties was comforting for them. The authors suggested that mental health services in Thailand should promote religious practices as a way of helping parents positively reframe events.

Studies also reported on the conscious efforts parents made to adjust the expectations they held about their child and the future (Kanungpiarn et al., 2021; Poonnotok et al., 2016; Raymond et al., 2017). Recognizing that their child may not lead a normal life, they re‐evaluated goals and lowered expectations. They also made a conscious effort to focus on positives, such as the good things in life (Pejlert, 2001), the love they felt for their child (McAuliffe et al., 2014), their child's strength (Wiens & Daniluk, 2009), the positive aspects of their child's illness and of their experience as parents (McCormack & McCann, 2015), and by noticing that the “positives outweigh the negatives” (Wade, 2006, p. 897).

The process of accepting the child's illness, adjusting expectations, and focusing on the positives afforded parents a renewed perspective on their lives and their futures. These conscious efforts to reframe their thinking, as well as religious practices and beliefs for some, were some of the ways in which parents were able to transcend their suffering.

#### Finding meaning

Despite their suffering, parents felt that they had no option other than to continue caring for their child. Finding meaning in their suffering and in their experiences afforded them the strength to continue, moving away from “ordinary caregiving experiences” (Donnelly, 2001, p. 299). Parents felt that they had found a purpose in life (McAuliffe et al., 2014), learned about themselves (Wiens & Daniluk, 2009), or learned to identify the real issues in life (Piuva & Brodin, 2020). Others found meaning, more directly, through their child, who had made them feel it was “all worth while” (Wade, 2006, p. 897). However, this concept was not universal. Parents across the studies were caring for children who were at different stages of diagnosis, treatment, and recovery; this may partly explain why only a small number of studies described experiences of finding meaning.

### Line of argument synthesis

**FIGURE 2:**
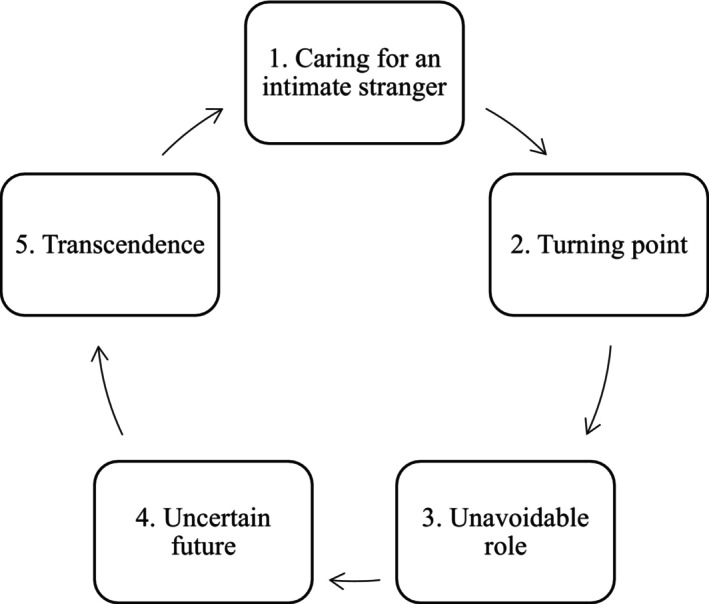
The psychological processes experienced by parents of children with mental illnesses.

All key concepts derived from the studies were illustrative of psychological processes experienced by parents who care for a child with a mental illness. This suggested that it would be possible to bring together these concepts and reciprocal translations in order to create a *line of argument synthesis* (Noblit & Hare, 1988); this goes beyond explaining the key concepts within the studies so as to formulate a grounded theory. In this way, the whole is greater than the sum of its parts (Noblit & Hare, 1988). The line of argument synthesis that follows depicts the temporal, subjective, and psychological experiences of parents and is presented diagrammatically (Figure 2).

## DISCUSSION

This review provided a meta‐ethnographic synthesis of 26 qualitative studies of parents' experiences of caring for a child with a mental illness. The proposed model depicts the psychological process experienced by parents when caring for a child with a mental illness.

This model is characterized by five stages, each depicting a transitional point in which parents re‐evaluate their experiences. Similar to Karp's (1994) five‐stage portrayal of the ongoing identity shifts of people experiencing depression, these stages involve a reassessment of one's identity and of one's life as a parent of a child with a mental illness.

The model is cyclical rather than linear. This reflects characteristics of the individual studies and the synthesis overall. In some studies, the parents had young children, while others had been caring for a (now adult) child with an enduring mental illness for decades. Children experienced periods of recovery followed by relapses, changes in psychological and pharmacological treatments, and transitions between treatment settings and stages of life; significant changes meant that some parents re‐experienced the early stages of the cycle. However, these parents had learned from their prior experiences, committed to their caregiving role, and found some meaning in their experiences so far. The ability to draw on previous experiences may have allowed them to engage with each stage with increased understanding and insight and pass through the stages more quickly.

Other nonlinear models have been used to understand experiences of coping and adjustment, such as bereavement. Stroebe and Schut's (1999) dual‐process model suggests that a person who is grieving oscillates between *loss orientation* (emotion‐focused coping) and *restoration orientation* (problem‐focused coping). The authors described a “dynamic, back‐and‐forth process” (Stroebe & Schut's, 1999, p. 216), in which oscillation between these orientations is necessary for optimal coping. The model proposed in this paper should also be considered dynamic and flexible, although until this model has been evaluated it will not be possible to say whether the stages are necessary for optimal adjustment. Kübler‐Ross (1969) proposed a five‐stage model of death, which she later applied to the process of grieving (Kübler‐Ross & Kessler, 2005). These five stages were not intended to denote a step‐by‐step or linear process through which everyone progresses but to help “frame and identify what we may be feeling” (Kubler‐Ross & Kessler, 2005, p. 5). Similarly, the model proposed in this paper is not intended to be prescriptive and should instead be used as a framework for beginning to understand these parents' complex subjective experiences. The cyclical component of the model is important but does not imply that all parents will pass through all stages or that all parents will cycle through them in the same way. Each stage of the model is expanded on below.

The five‐stage psychological process begins with parents noticing changes in their child. This first stage is characterized by painful emotions and shock and leads parents to feel as though they are caring for a stranger as their child is experienced as unrecognizable. Consequently, parents search for answers. The second stage is experienced as a turning point, marked by the realization that their child has a mental illness and by beginning to seek and receive help for their child. However, this perceived turning point does not manifest in the way that parents had anticipated. As parents engage with the healthcare system and learn more about their child's illness, they lose some of the hope that they had previously held. This leads to the third stage, in which parents face the unavoidable role of caring for their child. This role is experienced, at times, as impossibly difficult. The fourth stage is characterized by uncertainty; the future of both the child and the family feels unknown, and tentative moments of hope are offset by anxiety and fear. Parents, however, feel that they have no choice other than to continue caring for their child, whom they love. They find a way to cope; this final, fifth stage, depicts how parents attempt to reframe their thinking and find meaning in their experiences in order to transcend their suffering.

### Limitations

Despite there being only a relatively small number of studies eligible for inclusion in this review, the existing studies were conducted in a range of countries, drawing on a diverse population. However, given the small number conducted in each of the represented countries, it was not possible to draw conclusions based on geography or culture. Consequently, the proposed model neglects to consider intersectionality and how race, religion, education, healthcare practices, socioeconomic status, or other intersecting identities affect parents' experiences. It is possible, therefore, that the model presents a somewhat homogenized representation of diverse experiences.

The decision to include all studies uncovered by the systematic searches, regardless of quality, meant that the synthesis and proposed model are based on studies of variable quality. However, excluding weaker studies based on the quality appraisal would have significantly reduced the number of studies included in the review, making it more difficult to identify key concepts and draw conclusions. By highlighting the limitations of the individual studies in the quality appraisal, the results of the synthesis can be considered with these in mind.

### Implications

To test and evaluate the proposed model, future research should focus on some key areas. First, the validity of the model should be evaluated. The model could be tested initially with a small sample of parents, evaluating whether their experiences align with the proposed stages. Importantly, is there evidence to support the cyclical nature of the model? Furthermore, many of the studies included in the review concentrated on parents of children with psychotic disorders; is the model robust in the context of other mental illnesses?

Second, the utility of the model as a clinical framework to support clinicians working with families should be evaluated. Are clinicians able to reliably recognize which stage a parent is in, and does this help them to understand the experiences of the parent? The model highlights that parents may have difficult feelings about and toward their child; does this help clinicians to treat this with curiosity and empathy, recognizing that this is part of the process of coming to terms with a child's suffering? It will be important to ascertain whether the model can lead to helpful interventions for parents and whether this may have a correspondingly positive impact on the well‐being of the children.

Third, research should evaluate whether parents find the model useful. Does it normalize their experiences and/or help them formulate their difficulties and make sense of their feelings?

While this model is not intended to be prescriptive, it offers a framework for understanding parental experiences in the context of their children's mental illnesses. Research is now needed to evaluate whether the model can inform psychological interventions and other provisions of support for parents. The methodological and sampling issues in existing research, highlighted by the quality appraisal, should also be addressed by researchers moving forward in this field.

### Conclusion

To our knowledge, this is the first qualitative systematic review to meta‐synthesize research on the experiences of parents of children with mental illnesses. We found that experiences of subjective burden were described in all studies and proposed a five‐stage model that offers a new way of understanding the experiences of these parents, highlighting the complex psychological processes that they endure.

## Supporting information


Table S1.
