# Supplementary material for: Experiences of parents of children with mental illnesses: A systematic review and meta‐ethnographic synthesis
Source: Fam Process. 2024 Nov 24;64(1):e13087. doi: 10.1111/famp.13087 (PMC11803131; doi:10.1111/famp.13087)
Supplement: Supplementary file 1 — Table S1. [file FAMP-64-0-s001.docx]

Supplementary Material

Cross-Comparison of Studies by Concept

| **Key Concept** | **Transformation of the loved child** | **A psychological tsunami of emotions** | **Realisation** | **Getting help** | **Gendered caregiving** | **Commitment** | **An impossible task** | **Uncertain future** | **Reframing thinking** | **Finding meaning** |
| --- | --- | --- | --- | --- | --- | --- | --- | --- | --- | --- |
| Al Yahyaei et al., 2024 |  | * |  | * |  | * | * | * |  |  |
| Armitage et al., 2020 | * | * |  | * |  |  | * | * |  |  |
| Bai et al., 2020 | * | * | * | * |  |  | * | * |  |  |
| Darmi et al., 2017 | * | * | * | * |  | * | * | * |  | * |
| Donnelly, 2001 | * | * | * | * | * | * | * | * | * | * |
| Gok & Duyan, 2020 |  | * | * | * | * |  | * | * | * | * |
| Harden, 2005a | * | * | * | * |  | * | * | * | * |  |
| Harden, 2005b | * | * | * | * | * | * | * | * | * |  |
| Johansson, 2010 | * | * | * | * |  | * | * | * | * | * |
| Johansson, 2012 | * | * | * |  | * | * | * | * |  |  |
| Kalayci et al., 2022 |  | * | * | * |  |  | * | * |  |  |
| Kanungpiarn, 2021 | * | * | * | * |  | * | * | * | * |  |
| McAuliffe, 2014 | * | * | * | * |  | * | * | * | * | * |
| McCormack et al., 2015 | * | * |  | * | * | * |  | * | * |  |
| Mohr et al., 2001 | * | * | * | * |  | * | * | * |  |  |
| Pejlert, 2001 | * | * | * | * |  | * | * | * | * | * |
| Piuva & Brodin 2020 | * | * | * | * |  |  | * | * | * | * |
| Poonnotok et al., 2016 | * | * | * | * | * | * | * | * | * | * |
| Raymond et al., 2017 | * | * | * | * |  | * | * | * | * |  |
| Sarrió-Colas et al., 2022 | * |  | * | * | * |  | * | * |  | * |
| Stapley et al., 2016 | * | * | * | * |  | * | * | * |  |  |
| Svensson et al., 2013 | * | * |  | * | * |  | * | * | * |  |
| Thomson et al., 2014 | * |  | * | * |  |  | * |  |  |  |
| Tuck, 1997 | * | * | * | * |  | * | * | * | * | * |
| Wade, 2006 | * | * | * | * |  | * | * | * | * | * |
| Wiens & Daniluk, 2009 | * | * | * | * |  | * | * | * | * | * |
